# Corticosteroid-sparing effects of risankizumab versus ustekinumab in patients with moderately to severely active Crohn’s disease: post hoc results from the phase 3b SEQUENCE trial

**DOI:** 10.1093/ecco-jcc/jjag104

**Published:** 2026-07-21

**Authors:** Tim Raine, Silvio Danese, Stefan Schreiber, Xiang Gao, Stephen Hanauer, Joana Torres, Colla Cunneen, Kristina Kligys, Xiu Huang, Andrew Garrison, Ezequiel Neimark, Toni Anschutz, Jameson Crowley, Raymond K Cross

**Affiliations:** Department of Gastroenterology, Addenbrooke’s Hospital, Cambridge University Hospitals, Cambridge, United Kingdom; Department of Gastroenterology and Endoscopy, IRCCS Ospedale San Raffaele Hospital and Vita-Salute San Raffaele University, Milan, Italy; Department Internal Medicine I, Kiel University, University Hospital Schleswig-Holstein, Kiel, Germany; Department of Gastroenterology, The Sixth Affiliated Hospital of Sun Yat-sen University, Guangzhou, China; Division of Gastroenterology, Northwestern University, Chicago, IL, United States; Division of Gastroenterology, Hospital Beatriz Ângelo, Loures, Portugal; Faculdade de Medicina, Universidade de Lisboa, Lisbon, Portugal; Division of Gastroenterology, Hospital da Luz, Lisbon, Portugal; AbbVie Inc, North Chicago, IL, United States; AbbVie Inc, North Chicago, IL, United States; AbbVie Inc, North Chicago, IL, United States; AbbVie Inc, North Chicago, IL, United States; AbbVie Inc, North Chicago, IL, United States; AbbVie Inc, North Chicago, IL, United States; AbbVie Inc, North Chicago, IL, United States; Melissa L. Posner Institute for Digestive Health and Liver Disease at Mercy Medical Center, Baltimore, MD, United States

**Keywords:** clinical trials, endoscopy, quality of life, socio-economical and psychological endpoints

## Abstract

**Background:**

This post hoc analysis evaluated the corticosteroid-sparing effects of risankizumab (RZB) versus ustekinumab (UST) in patients with moderate to severe Crohn’s disease (CD) with prior inadequate response or intolerance to ≥ 1 anti-tumor necrosis factor (TNF) therapy.

**Methods:**

SEQUENCE (NCT04524611) was an open-label, multicenter, randomized, efficacy assessment-blinded study. Patients were randomized 1:1 to receive RZB (intravenous [IV] 600 mg induction dose at weeks 0, 4, and 8, then a subcutaneous [SC] 360 mg maintenance dose every 8 weeks [Q8w] starting at week 12) or UST (single weight-based IV induction dose, then a SC 90 mg maintenance dose Q8w starting at week 8) up to week 48. Patients tapered corticosteroids beginning at week 2. Achievement of clinical, endoscopic, and quality of life outcomes without concomitant use of steroids (“corticosteroid-free”) and safety were assessed.

**Results:**

In patients taking corticosteroids at baseline, response rate differences by the week 24 and 48 timepoints with RZB versus UST were 10.5% and 25.4% (*P* ≤ .01) for corticosteroid-free clinical remission per CD activity index and 20.4% (*P* ≤ .001) and 29.2% (*P* ≤ .01) per stool frequency/abdominal pain score, and 23.0% and 20.2% (both *P* ≤ .01) for corticosteroid-free endoscopic remission. Similar results were also observed for additional corticosteroid-free endpoints, including endoscopic, composite (clinical + endoscopic), and quality of life outcomes. Numerically higher exposure-adjusted event rates of serious infections and hypersensitivity with RZB and UST were observed with corticosteroid use.

**Conclusion:**

In patients with CD refractory to anti-TNF therapy, higher rates of corticosteroid-free clinical, endoscopic, and quality of life outcomes were achieved with RZB versus UST. Both treatments were well-tolerated.

**Clinical trial registration number:**

NCT04524611

## Graphical abstract

**Graphical Abstract:**
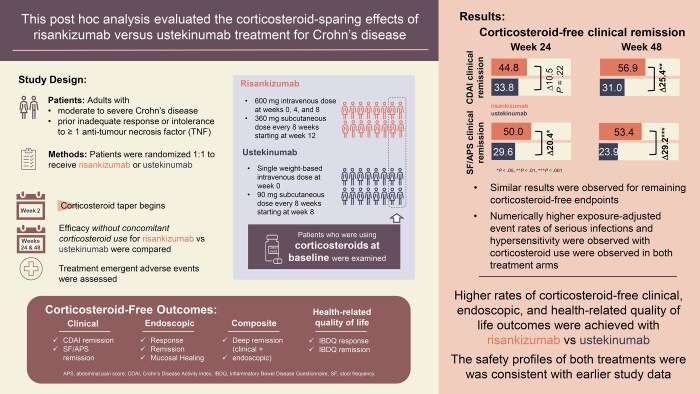

## 1. Introduction

Crohn’s disease (CD) is a chronic, progressive inflammatory bowel disease (IBD) characterized by unpredictable flares followed by periods of remission. If left untreated, CD can result in complications that require hospitalization and surgery and may lead to disability.^1^

Historically, corticosteroids have been used to treat symptom flares and induce clinical remission in patients with IBD; they are not, however, effective for the maintenance of long-term remission. Moreover, prolonged or repeated use of corticosteroids can lead to serious short-term and long-term adverse effects, as well as dependency.^2–6^ These adverse effects are associated with increased healthcare resource utilization and costs, leading to greater economic burden on the patient.^7^ Current treatment guidelines now recommend the early introduction of an advanced therapy (biologics, small molecules) instead of delaying their use until after failure of 5-aminosalicylates and/or corticosteroids. Nevertheless, corticosteroid therapy remains the most common first line of treatment for patients with moderate to severe CD, with rates of corticosteroid use remaining steady despite the availability of advanced therapies.^8–17^ A better understanding of the corticosteroid-sparing properties of advanced therapies and their comparative effectiveness is needed to minimize steroid exposure and optimize disease control.

Risankizumab (RZB) is a high-affinity humanized IgG1 monoclonal antibody that selectively binds to the p19 subunit of human interleukin (IL)-23 cytokine and inhibits its interaction with the IL-23 receptor. Post hoc analysis of data from the pivotal phase 3 FORTIFY study demonstrated the corticosteroid-sparing effects of RZB by showing greater discontinuation of corticosteroids with RZB than placebo.^18^^,^^19^ In addition, rates of corticosteroid-free clinical remission per stool frequency (SF)/abdominal pain score (APS) and per CD activity index (CDAI), as well as corticosteroid-free endoscopic response, were greater with RZB than placebo.^18^ The phase 3b head-to-head SEQUENCE trial compared the efficacy and safety of RZB and ustekinumab (UST), an IL-23 inhibitor that targets both IL-12 and IL-23 via their common p40 subunit, in patients with moderate to severe CD who demonstrated inadequate response or intolerance to ≥ 1 anti-tumor necrosis factor (TNF) therapies.^20^ In this study, achievement of the secondary endpoints of corticosteroid-free clinical remission and corticosteroid-free endoscopic remission with RZB were superior to UST following maintenance treatment.^20^ Here, we further examined the corticosteroid-sparing effects of RZB versus UST over 48 weeks of treatment in patients taking corticosteroids at baseline in the SEQUENCE study.

## 2. Methods

### Trial design and oversight

The SEQUENCE (NCT04524611) phase 3b, multicenter, open-label, randomized, efficacy assessment-blinded trial was performed at 187 sites in 28 countries.^20^ Part 1 of the SEQUENCE study examined the efficacy and safety of RZB versus UST over a period of 48 weeks in adults with moderate to severe CD (defined as a baseline CDAI from 220 to 450, average daily SF ≥ 4 and/or average daily APS ≥ 2, and simple endoscopic score for CD [SES-CD] ≥ 5 [≥4 for isolated ileal disease]) who had previously experienced intolerance or inadequate response to at least 1 anti-TNF therapy. Inadequate response was defined as signs and symptoms of persistently (in the opinion of the investigator) active disease despite a history of 1 or more of the following: at least one 6-week induction regimen of infliximab (≥5 mg/kg IV at weeks 0, 2, and 6), at least one 4-week induction regimen of adalimumab (single 160 mg SC dose at week 0, followed by 80 mg SC dose at week 2 [or one 80 mg SC dose at week 0, followed by 40 mg SC dose at week 2, in countries where this dosing regimen is approved]), and/or at least one 4-week induction regimen of certolizumab pegol (400 mg SC at weeks 0, 2, and 4), as well as recurrence of symptoms during scheduled maintenance dosing following prior clinical benefit of the above anti-TNF agents. Patients who discontinued anti-TNF agents for reasons other than inadequate response as defined above (eg, change of insurance) or intolerance were not eligible to participate in SEQUENCE. Patients could not have received infliximab, certolizumab pegol, and/or adalimumab (including biosimilars) within 8 weeks prior to baseline or had received any other approved biologic and/or targeted small molecules, including RZB or UST.

Patients were randomized 1:1 to receive RZB or UST, and randomization was stratified according to the number of previously received anti-TNF therapies (1 or >1) and glucocorticoid use at baseline (yes or no). Patients received the recommended dosage of RZB (600 mg intravenous [IV] dose at weeks 0, 4, and 8, followed by a 360 mg subcutaneous [SC] dose every 8 weeks from week 12 to week 48) or UST (a single weight-based IV dose [patients weighing ≤ 55 kg received 260 mg, patients weighing >55-85 kg received 390 mg, and patients weighing >85 kg received 520 mg] at week 0 followed by a 90 mg SC dose every 8 weeks, starting at week 8).

### Corticosteroid taper

Patients taking oral corticosteroids at the following maximum doses were eligible for enrollment if they were on the current treatment course ≥14 days and were on a stable dose for ≥7 days, prior to baseline: budesonide ≤9 mg/day, beclomethasone ≤5 mg/day, prednisone or equivalent ≤20 mg/day. Decreasing doses of corticosteroids were prohibited during the first 2 weeks, except in the event of moderate-to-severe treatment-related toxicities and after discussion with the sponsor. A mandatory glucocorticoid taper was initiated at week 2 according to a protocol-specified tapering schedule (Table S1). If a patient experienced inadequate response during the corticosteroid taper, the corticosteroid dose could be increased per the investigator’s discretion up to the dose used at baseline. Patients for whom the maximum steroid dose exceeded the dose used at baseline were considered nonresponders for efficacy assessments from that point forward but continued to be evaluated in the safety population. For patients who underwent initiation or dose-escalation of CD-related corticosteroids, values at or after the initiation or dose-escalation of CD-related corticosteroids were excluded from the analysis; patients were considered nonresponders at or after the initiation or dose-escalation of CD-related corticosteroids.

### Ethics

This clinical trial was conducted in accordance with the operations manual, protocol, International Council for Harmonization guidelines, and applicable guidelines and regulations governing ethical principles and study conduct originating in the Declaration of Helsinki. An independent ethics committee/institutional review board ensured the ethical, scientific, and medical appropriateness of the study before it was conducted and approved all relevant documentation. This clinical trial was prospectively registered at Clinicaltrials.gov (NCT04524611). Written informed consent was obtained from patients before enrollment.

### Assessments

For patients using corticosteroids at baseline, mean corticosteroid dose was evaluated in patients receiving RZB versus UST. In addition, efficacy was evaluated in patients using corticosteroids at baseline (yes/no) by the achievement of corticosteroid-free clinical and endoscopic outcomes, using a definition of “no corticosteroids at the corresponding study visit.” All corticosteroid-free outcomes post-baseline were evaluated at weeks 24 and 48 of the maintenance period. Endpoints included clinical remission (per 2 definitions, CDAI [<150] and SF/APS [average daily SF ≤2.8 and daily APS ≤1 and both not worse than baseline]), endoscopic response (>50% decrease from baseline in SES-CD or ≥2-point reduction from baseline for patients with isolated ileal disease and baseline SES-CD of 4), endoscopic remission (SES-CD ≤4 and ≥2-point reduction versus baseline and no subscore >1 in any individual variable), deep remission (a composite of CDAI clinical remission and endoscopic remission), and mucosal healing (SES-CD ulcerated surface subscore of 0 in patients with SES-CD ulcerated surface subscore ≥1 at baseline as scored by a central reviewer). Corticosteroid-free CDAI clinical remission and corticosteroid-free endoscopic remission were also further evaluated using a more stringent definition of corticosteroid discontinuation: no corticosteroid use for ≥90 days relative to the corresponding study visit (week 48).

In addition to clinical and endoscopic endpoints, corticosteroid-free health-related quality of life (HRQoL) was also assessed. HRQoL endpoints included inflammatory bowel disease questionnaire (IBDQ) response (increase in IBDQ total score ≥16 points from baseline) and corticosteroid-free IBDQ remission (IBDQ total score ≥170 points).

Safety by baseline corticosteroid use was evaluated. Treatment-emergent adverse events (TEAEs) were tabulated using the Medical Dictionary for Regulatory Activities (version 26.0) system organ class and preferred terms. Independent committees were established to adjudicate any observed systemic hypersensitivity and anaphylactic events and cardiac and cerebrovascular events; these committees were blinded to treatment allocation.

### Statistical analysis

All efficacy analyses were performed post hoc in randomized patients in the intention-to-treat population (patients in the selected RZB dose regimen [600 mg IV/360 mg SC] group who received ≥1 dose of RZB or UST in the SEQUENCE trial). Categorical endpoints were analyzed using nonresponder imputation incorporating multiple imputation to handle missing data due to COVID-19 and/or geopolitical conflict. The proportion of responders and associated 95% confidence intervals (CIs) were reported for each treatment group. The adjusted risk difference between RZB and UST, associated 95% CI, and nominal *P-*value for each efficacy endpoint were also reported. Cochran–Mantel–Haenszel The 95% CI for the adjusted difference and *P-*value were calculated according to the (CMH) test adjusted for randomization strata for the comparison of 2 treatment groups. For analyses on the subpopulation of patients taking corticosteroids at baseline, baseline corticosteroid status was removed as a factor from the CMH test. Patients who had new corticosteroid initiation or a corticosteroid dose increase above the baseline dose were considered as nonresponders for all timepoints after the event occurred.

Safety data analyzed in this study were presented as exposure-adjusted event rates (events [E] per 100 patient-years [PY]) and were analyzed in all randomized patients who received ≥1 dose of RZB or UST. TEAEs and TEAEs of special interest were defined as events that began either on or after the first dose of the study drug in SEQUENCE and until the first dose of study drug (RZB) in the open-label long-term extension (ie, SEQUENCE Part 2), if the patient was enrolled in Part 2, or within 140 days after the last dose administration of the study drug (UST or RZB) in Part 1 if the patient did not participate in Part 2. Safety data before the cutoff date of July 12, 2023, or the week 52 dosing date (the first dose date of Part 2), whichever occurred earlier, were evaluated in this analysis.

## 3. Results

### Patients

Of the 520 patients in the intention-to-treat population randomized and treated in Part 1 of SEQUENCE, 129 patients (24.8%; RZB, *n* = 58; UST, *n* = 71) were taking corticosteroids at baseline; of these patients, a greater proportion who received RZB treatment in Part 1 were on prednisone/prednisolone (RZB, 39.4%/22.5%; UST, 31.0%/15.5%) at baseline, while a greater proportion of patients who received UST were on budesonide (UST, 27.6%; RZB, 18.3%). The mean (standard deviation) daily prednisone-equivalent corticosteroid dose at baseline for patients in the RZB arm were 21.4 (12.9) mg/day and 19.5 (10.7) mg/day for patients in the UST arm. Patient demographics and disease characteristics at baseline were generally similar between patients who did and did not use corticosteroids at baseline in both the RZB and UST groups, with the exception of immunomodulator use, which was greater in patients who were not taking corticosteroids at baseline (14.2% RZB, 22.7% UST) versus patients who were taking corticosteroids at baseline (10.3% RZB, 4.2% UST; Table 1).

**Table 1.** Baseline demographics and disease characteristics in the intention-to-treat population by baseline corticosteroid use.

Parameter	Corticosteroid use		No corticosteroid use	
	Risankizumab	Ustekinumab	Risankizumab	Ustekinumab
	*n* = 58	*n* = 71	*n* = 197	*n* = 194
**Female, *n* (%)**	27 (46.6)	37 (52.1)	92 (46.7)	97 (50.0)
**Age, years, mean (SD)**	37.6 (11.7)	39.0 (13.9)	38.2 (13.6)	38.0 (13.8)
**Weight, kg, mean (SD)**	67.2 (16.5)	70.7 (19.6)	69.4 (18.3)	71.5 (19.4)
**Corticosteroid use, *n* (%)**			0	0
** Budesonide**	16 (27.6)	13 (18.3)	−	−
** Deflazacort**	0	1 (1.4)	−	−
** Dexamethasone**	0	0	−	−
** Meprednisone**	2 (3.4)	4 (5.6)	−	−
** Methylprednisolone**	7 (12.1)	8 (11.3)	−	−
** Prednisolone**	9 (15.5)	16 (22.5)	−	−
** Prednisone**	18 (31.0)	28 (39.4)	−	−
** Unspecified**	6 (10.3)	1 (1.4)	−	−
**Daily prednisone equivalent dose, median (IQR)**	21.4 (12.9)	19.5 (10.7)	–	–
**Immunomodulator use, *n* (%)**	6 (10.3)	3 (4.2)	28 (14.2)	44 (22.7)
**Prior anti-tumor necrosis factor failure, *n* (%)**				
** 0**	0	0	1 (0.5)	0
** 1**	44 (75.9)	50 (70.4)	151 (76.6)	154 (79.4)
** ≥1**	14 (24.1)	21 (29.6)	45 (22.8)	40 (20.6)
**CD duration, mean (SD)**	9.3 (9.0)	9.8 (9.6)	9.5 (7.4)	9.3 (8.3)
**Disease location, *n* (%) **				
** Ileal only**	3 (5.2)	13 (18.3)	39 (19.8)	32 (16.5)
** Colonic only**	26 (44.8)	31 (43.7)	76 (38.6)	75 (38.7)
** Ileal-colonic**	29 (50.0)	27 (38.0)	82 (41.6)	87 (44.8)
**FCP, mg/kg, median (Q1, Q3)**	*n* = 47; 1043.0 (369.0, 3505.0)	*n* = 58; 2135.0 (1236.0, 3361.0)	*n* = 160; 1001.5 (340.5, 2273.5)	*n* = 157; 1247.0 (348.0, 2534.0)
**hsCRP, mg/L, median (Q1, Q3)**	*n* = 57; 7.50 (2.70, 32.30)	*n* = 66; 10.25 4.70, 41.70)	*n* = 189; 8.30 (3.70, 22.40)	*n* = 191; 9.30 (3.10, 25.00)
**CDAI, mean (SD)**	*n* = 57; 329.4 (73.5)	314.1 (59.1)	*n* = 194; 303.6 (56.7)	*n* = 192; 308.7 (64.0)
**SES-CD, mean (SD)**	*n* = 57; 15.1 (6.24)	14.4 (7.56)	13.0 (7.23)	14.0 (7.35)
**Average daily SF, mean (SD)**	*n* = 57; 6.5 (3.3)	5.9 (2.7)	*n* = 194; 4.9 (2.7)	*n* = 192; 5.4 (2.3)
**Average daily APS, mean (SD)**	*n* = 57; 2.0 (0.5)	2.0 (0.5)	*n* = 194; 2.0 (0.6)	*n* = 192; 2.0 (0.9)

Abbreviations: APS, abdominal pain score; CD, Crohn’s disease; CDAI, CD activity index; FCP, fecal calprotectin; hsCRP, high-sensitivity C reactive protein; IQR, interquartile range; SES-CD, simple endoscopic score for CD; SF, stool frequency.

### Efficacy

**Figure 1.**
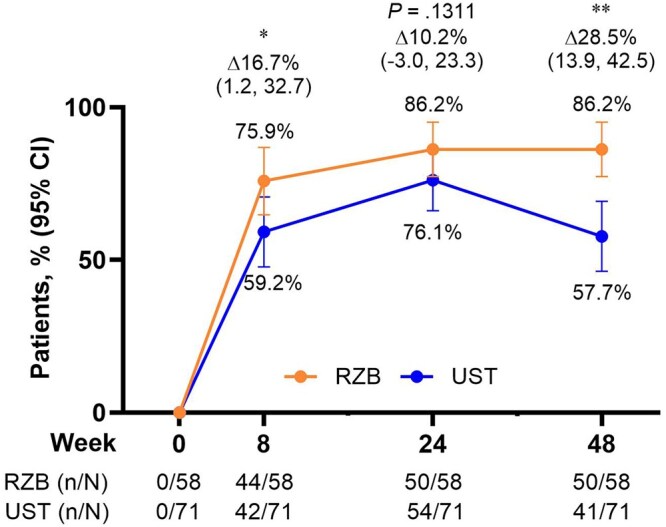
Discontinuation of corticosteroids in patients taking corticosteroids at baseline. CI, confidence interval; RZB, risankizumab; UST, ustekinumab. *P ≤ .05; **P ≤ .001. *P*-values are nominal.

By week 8, more patients had discontinued corticosteroids in the RZB arm (75.9% [*n*/*N* = 44/58]) vs the UST arm (59.2% [42/71]; adjusted difference, 16.7 percentage points [95% CI, 1.2-32.7], *P* < .05) (Figure 1). Although a similar proportion of patients in either treatment group discontinued corticosteroids by week 24, more patients treated with RZB versus UST discontinued corticosteroids by week 48 (86.2% [50/58] vs 57.7% [41/71]; adjusted difference, 28.5 percentage points [95% CI, 13.9-42.5], *P* < .001) (Figure 1).

**Figure 2.**
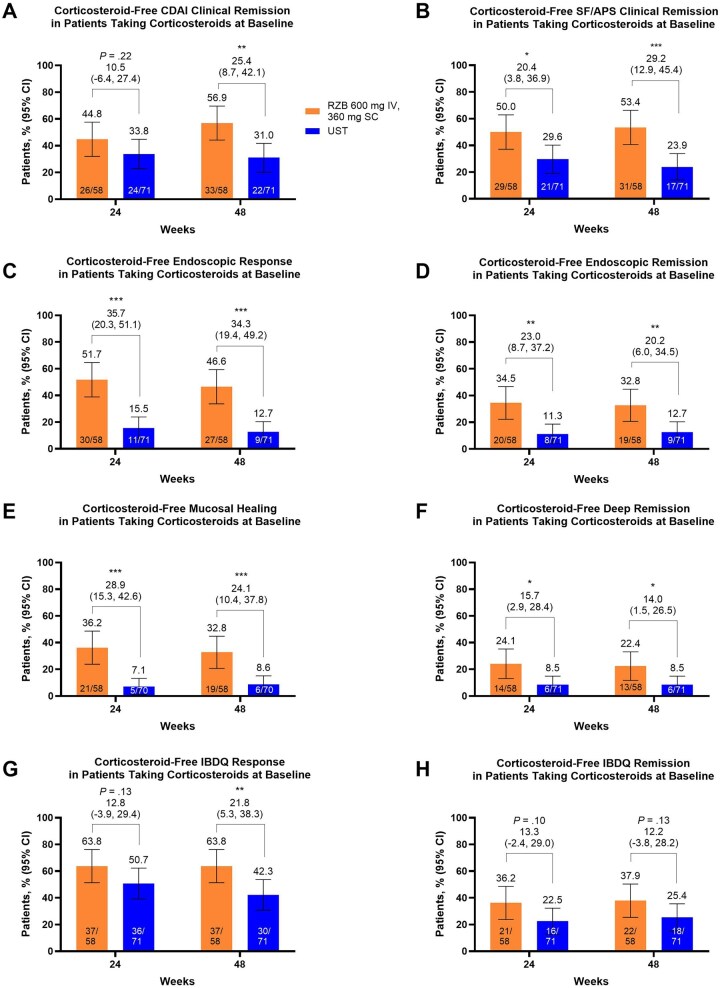
Corticosteroid-free clinical, endoscopic, and quality of life outcomes by weeks 24 and 48 of SEQUENCE in patients taking corticosteroids at baseline. APS, abdominal pain score; CDAI, Crohn’s disease activity index; CI, confidence interval; IBDQ, inflammatory bowel disease questionnaire; IV, intravenous; RZB, risankizumab; SC, subcutaneous; SES-CD, simple endoscopic score for Crohn’s disease; SF, stool frequency; UST, ustekinumab. Values above bars represent % (95% CI). *P ≤ .05; **P ≤ .01; ***P ≤ .001. *P*-values are nominal. Corticosteroid-free, no corticosteroids at the corresponding study visit; CDAI clinical remission, CDAI < 150; SF/APS clinical remission, average daily SF ≤2.8 and daily APS ≤1 and both not worse than baseline; endoscopic response, >50% decrease from baseline in SES-CD or ≥2-point reduction from baseline for patients with isolated ileal disease and baseline SES-CD of 4; endoscopic remission, SES-CD ≤4 and ≥2-point reduction vs baseline and no subscore >1 in any individual variable; deep remission, CDAI clinical remission plus endoscopic remission; mucosal healing, SES-CD ulcerated surface subscore of 0 in patients with SES-CD ulcerated surface subscore ≥1 at baseline as scored by a central reviewer; IBDQ response, increase in IBDQ total score ≥16 points from baseline; IBDQ remission, IBDQ total score ≥170 points.

Among patients taking corticosteroids at baseline, higher rates of corticosteroid-free clinical remission were achieved by patients in the RZB arm versus the UST arm. By week 24, corticosteroid-free CDAI clinical remission was achieved by 44.8% (26/58) of patients in the RZB arm versus 33.8% (24/71) of patients in the UST arm (adjusted difference, 10.5 percentage points [95% CI, −6.4 to 27.4], *P* = .22) (Figure 2A); by week 48, this difference was greater (56.9% [33/58] of patients in the RZB arm vs 31.0% [22/71] of patients in the UST arm; adjusted difference, 25.4 percentage points [95% CI, 8.7-42.1], *P* < .01). For corticosteroid-free SF/APS clinical remission, the response rate differences for RZB were higher than UST by both week 24 (RZB: 50.0% [29/58] vs UST: 29.6% [21/71]; adjusted difference, 20.4 percentage points [95% CI, 3.8 to 36.9], *P* < .05) and week 48 (RZB: 53.4% [31/58] vs UST: 23.9% [17/71]; adjusted difference, 29.2 percentage points [95% CI, 12.9-45.4], *P* < .001) (Figure 2B).

Additionally, among patients taking corticosteroids at baseline, rates of efficacy by week 24 and week 48 were greater with RZB versus UST for corticosteroid-free endoscopic response (week 24: 51.7% [30/58] vs 15.5% [11/71], adjusted difference, 35.7 percentage points [95% CI, 20.3-51.1], *P* < .001; week 48: 46.6% [27/58] vs 12.7% [9/71], adjusted difference, 34.3 percentage points [95% CI, 19.4-49.2], *P* < .001; Figure 2C) and corticosteroid-free endoscopic remission (week 24: 34.5% [20/58] vs 11.3% [8/71], adjusted difference, 23.0 percentage points [95% CI, 8.7-37.2], *P* < .01; week 48: 32.8% [19/58] vs 12.7% [9/71], adjusted difference, 20.2 percentage points [95% CI, 6.0-34.5], *P* < .01; Figure 2D). Higher rates were also observed in the RZB arm versus UST arm when evaluating more stringent endoscopic endpoints, including corticosteroid-free mucosal healing (week 24: 36.2% [21/58] vs 7.1% [5/70], adjusted difference, 28.9 percentage points [95% CI, 15.3-42.6], *P* < .001; week 48: 32.8% [19/58] vs 8.6% [6/70]; adjusted difference, 24.1 percentage points [95% CI, 10.4-37.8], *P* < .001; Figure 2E) and corticosteroid-free deep remission (week 24: 24.1% [14/58] vs 8.5% [6/71], adjusted difference, 15.7 percentage points [95% CI, 2.9-28.4], *P* < .05; week 48: 22.4% [13/58] vs 8.5% [6/71], adjusted difference, 14.0 percentage points [95% CI, 1.5-26.5], *P* < .05; Figure 2F).

With respect to HRQoL outcomes, a numerically greater proportion of patients demonstrated improved symptoms and daily functioning with RZB versus UST as reflected by the achievement of corticosteroid-free IBDQ response (Figure 2G) by week 24 (63.8% [37/58] vs 50.7% [36/71], adjusted difference, 12.8 percentage points [95% CI, −3.9 to 29.4], *P* = .13), and, by week 48, a significantly greater proportion of patients achieved corticosteroid-free IBDQ response with RZB versus UST (63.8% [37/58] vs 42.3% [30/71], adjusted difference, 21.8 percentage points [95% CI, 5.3-38.3], *P* < .01). Moreover, a numerically greater proportion of patients also demonstrated corticosteroid-free IBDQ remission (Figure 2H), indicative of a HRQoL similar to healthy individuals, with RZB versus UST. When examining these endpoints for efficacy with RZB versus UST for all patients, irrespective of corticosteroid use at baseline, RZB-treated patients achieved higher rates of efficacy for all corticosteroid-free endpoints by both timepoints (Figure S2, see online supplementary material for a color version of this figure).

**Figure 3.**
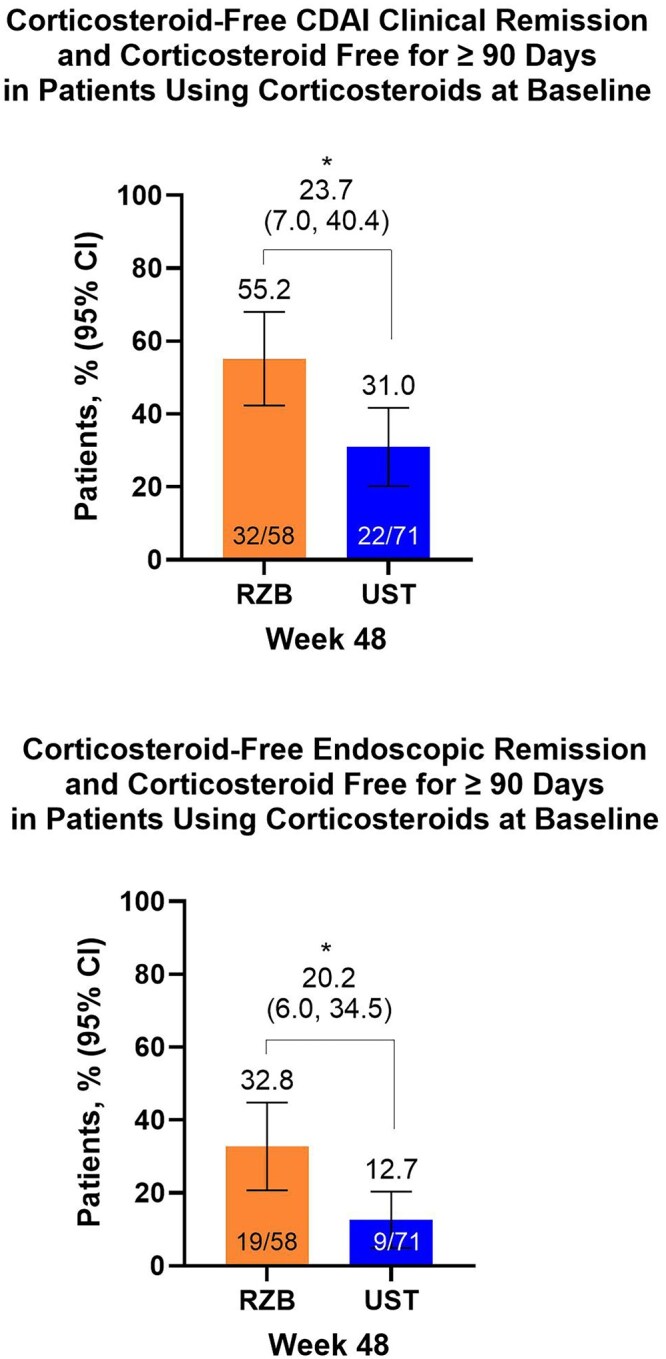
Clinical remission and endoscopic remission and corticosteroid-free for 90 days at week 48 in SEQUENCE. APS, abdominal pain score; CDAI, Crohn’s disease activity index; CI, confidence interval; RZB, risankizumab; SES-CD, simple endoscopic score for CD; SF, stool frequency; UST, ustekinumab. Values above bars represent % (95% CI). *P ≤ .01. *P*-values are nominal. CDAI clinical remission, CDAI <150; SF/APS clinical remission, average daily SF ≤2.8 and daily APS ≤1 and both not worse than baseline; endoscopic remission, SES-CD ≤4 and ≥2-point reduction vs baseline and no subscore >1 in any individual variable.

Similar results for corticosteroid-free clinical remission and corticosteroid-free endoscopic remission by week 48 were observed when corticosteroid-free was defined as no corticosteroid use for at least 90 days (Figure 3), indicating that most patients that were not taking steroids at week 48 had been corticosteroid-free for at least 90 days.

### Safety

During part 1, the exposure-adjusted event rates of TEAEs were generally similar between the treatment groups, regardless of baseline corticosteroid use (Table 2). However, the proportion of patients with TEAEs leading to study drug discontinuation was numerically higher in patients who were using corticosteroids at baseline and who received UST versus patients who were using corticosteroids at baseline and who received RZB (Table S2). The exposure-adjusted rates of TEAEs of special interest were generally low with both RZB and UST, regardless of corticosteroid use at baseline, except serious infections, herpes zoster, hypersensitivity, and injection site reactions, which were numerically higher in patients with corticosteroid use at baseline versus those without, and hepatic events, which were numerically lower in patients with corticosteroid use at baseline versus those without.

**Table 2.** Overview of treatment-emergent adverse events in the safety population by baseline corticosteroid use.

Parameter, E (E/100 PY)	Corticosteroid use			No corticosteroid use		
	Risankizumab	Ustekinumab	95% CI for Treatment Difference	Risankizumab	Ustekinumab	95% CI for Treatment Difference
	*n* = 62 PYs = 60.9	*n* = 71 PYs = 69.7	Risankizumab versus Ustekinumab	*n* = 200 PYs = 196.7	*n* = 194 PYs = 200.1	Risankizumab versus Ustekinumab
**Any TEAEs**	193 (317.1)	206 (295.4)	21.7 (−38.5, 81.9)	686 (348.7)	557 (278.3)	70.4 (35.5, 105.2)
** Severe TEAE**	15 (24.6)	26 (37.3)	−12.6 (−31.6, 6.4)	45 (22.9)	56 (28.0)	−5.1 (−15.0, 4.8)
** Serious TEAEs**	11 (18.1)	21 (30.1)	−12.0 (−28.8, 4.7)	25 (12.7)	43 (21.5)	−8.8 (−16.9, −0.7)
** TEAEs leading to study drug discontinuation**	2 (3.3)	6 (8.6)	−5.3 (−13.6, 2.9)	8 (4.1)	8 (4.0)	0.1 (−3.9, 4.0)
** Death**	0	0	0	0	0	0
**TEAEs of special interest**						
** Adjudicated MACE**	0	0	0	0	1 (0.5)	−0.5 (−1.5, 0.5)
** Extended MACE**	0	0	0	0	1 (0.5)	−0.5 (−1.5, 0.5)
** Serious infections**	3 (4.9)	7 (10.0)	−5.1 (−14.4, 4.2)	7 (3.6)	7 (3.5)	0.1 (−3.6, 3.8)
** Opportunistic infections (excluding tuberculosis and herpes zoster)**	1 (1.6)	0	1.6 (−1.6, 4.9)	0	0	0
** Herpes zoster**	1 (1.6)	1 (1.4)	0.2 (−4.1, 4.5)	0	0	0
** Malignant tumors**	0	0	0	1 (0.5)	1 (0.5)	0.0 (−1.4, 1.4)
** NMSC**	0	0	0	1 (0.5)	0	0.5 (−0.5, 1.5)
** Malignancies excluding NMSC**	0	0	0	0	1 (0.5)	−0.5 (−1.5, 0.5)
** Hypersensitivity**	12 (19.7)	9 (12.9)	6.8 (−7.2, 20.8)	25 (12.7)	23 (11.5)	1.2 (−5.6, 8.1)
** Hepatic events**	1 (1.6)	3 (4.3)	−2.7 (−8.5, 3.2)	25 (12.7)	20 (10.0)	2.7 (−3.9, 9.3)
** Injection site reactions**	2 (3.3)	2 (2.9)	0.4 (−5.6, 6.5)	3 (1.5)	6 (3.0)	−1.5 (−4.4, 1.5)

Abbreviations: CI, confidence interval; E, events; MACE, major adverse cardiovascular event; NMSC, nonmelanoma skin cancer; PY, patient-years; TEAE, treatment-emergent adverse event; UST, ustekinumab.

No events of active tuberculosis, serious hypersensitivity, or adjudicated anaphylactic reaction.

## 4. Discussion

Symptomatic response and remission and endoscopic healing are important treatment targets for patients with moderate to severe CD.^21^ Although corticosteroids are effective for inducing remission and managing flares, they lack effectiveness for maintaining clinical remission or achieving endoscopic healing, and their long-term use is associated with dependency and significant side effects. Current IBD treatment guidelines now recommend limiting patient corticosteroid exposure and emphasize the importance of achieving corticosteroid-free clinical and endoscopic outcomes.^12^^,^^16^

Advanced therapies have been found to be more effective when used earlier in the course of CD.^22–24^ For example, recent evidence has shown that compared to conventional step-up treatment, a top-down strategy (immunomodulator + infliximab) led to substantially better outcomes, including corticosteroid-free remission after 1 year.^24^ While anti-TNFs are the first biological agents for the treatment of CD, loss of response over 12 months occurs in a substantial proportion of patients.^25^ The availability of newer advanced therapies (eg, vedolizumab [VDZ], an anti-α4β7 integrin monoclonal antibody approved by the U.S. Food and Drug Administration [FDA] for CD in 2014; UST, FDA approved for treatment of CD in 2016; and, more recently, specific anti-IL23 p19 agents, such as RZB, mirikizumab, and guselkumab, FDA approved for treatment of CD in 2022, 2025, and 2025, respectively) provide alterative treatment options to anti-TNFs.^25^ To date, retrospective studies comparing UST versus VDZ have generally shown UST to be more effective than VDZ for achieving corticosteroid-free remission.^26^ However, additional comparative safety and efficacy assessments, such as those reported in this post hoc analysis, are needed to inform treatment decision-making.

Although management of corticosteroids in clinical trials in IBD is often left to investigator judgment, SEQUENCE included an early mandatory corticosteroid taper, allowing a protocolized comparison in the use of corticosteroids between the evaluated treatments.^20^ The efficacy of RZB was previously demonstrated to be significantly better than UST across numerous clinical and endoscopic endpoints, including week 48 corticosteroid-free clinical remission and corticosteroid-free endoscopic remission.^20^ In this post hoc analysis, we examined corticosteroid discontinuation over time and the achievement of corticosteroid-free clinical, endoscopic, and HRQoL endpoints with RZB versus UST by weeks 24 and 48 in patients who were taking corticosteroids at baseline, as well as in all patients. Our results demonstrated that RZB was more effective than UST for meeting these important treatment goals, including discontinuation of corticosteroid use as early as week 8, and achievement of the corticosteroid-free endpoints of clinical remission (per CDAI and SF/APS), endoscopic response, endoscopic remission, mucosal healing, and deep remission, as well as the HRQoL endpoint of IBDQ response, as early as week 24 in patients taking corticosteroids at baseline. In all patients, these corticosteroid-free endpoints, plus IBDQ remission, were also achieved by a greater proportion of patients with RZB versus UST by weeks 24 and 48. The proportion of patients who achieved clinical and HRQoL endpoints with RZB were generally similar between all patients and patients taking corticosteroids at baseline; for endoscopic endpoints and deep remission, a similar or greater proportion of patients who were taking corticosteroids at baseline achieved these endpoints with RZB compared to all patients. Taken together, these findings show greater efficacy of RZB versus UST in all patients and in patients who have received prior corticosteroid therapy and underscore the corticosteroid-sparing potential of RZB.

Both RZB and UST were well-tolerated in patients with moderate to severe CD, with generally similar exposure-adjusted events rates of TEAEs regardless of baseline corticosteroid use and consistent with the primary analyses.^19^^,^^27^^,^^28^ Corticosteroid use was, however, associated with higher rates of serious infections, herpes zoster, hypersensitivity, and injection site reactions, which were numerically higher in patients with corticosteroid use at baseline versus those without, regardless of treatment. Hepatic events were numerically lower in patients with corticosteroid use at baseline versus those without.

A strength of this analysis is the prospective randomization of patients in SEQUENCE, which was stratified by baseline corticosteroid use, enabling the number of patients with and without corticosteroid use to be comparable between treatments. However, most patients were not taking corticosteroids at baseline, which limited the number of patients available for this subgroup analysis and resulted in increased variability in efficacy rates. This limitation, therefore, should be considered with respect to the interpretation of the results presented herein. In addition, this study was open label, so the patient’s knowledge of their treatment may have influenced subjective measures such as the CDAI and IBDQ. Similar differences between RZB and UST response rates, however, were observed for objective endoscopic endpoints, corroborating these findings.

In summary, greater rates of corticosteroid-free clinical and/or endoscopic and HRQoL outcomes were achieved with RZB versus UST in all patients, as well as in patients taking corticosteroids at baseline. RZB and UST were well-tolerated regardless of baseline corticosteroid use, with safety profiles that were generally similar to those observed in the primary analyses.^19^^,^^27^^,^^28^ The greater relative effectiveness of RZB versus UST for corticosteroid discontinuation and for achieving and maintaining corticosteroid-free outcomes in patients with moderate to severe CD underscore the emerging value of RZB as a corticosteroid-sparing therapy.

## Supplementary Material

jjag104_Supplementary_Data

## Data Availability

AbbVie is committed to responsible data sharing regarding the clinical trials we sponsor. This includes access to anonymized, individual, and trial-level data (analysis data sets), as well as other information (eg, protocols, clinical study reports, or analysis plans), as long as the trials are not part of an ongoing or planned regulatory submission. This includes requests for clinical trial data for unlicensed products and indications. These clinical trial data can be requested by any qualified researchers who engage in rigorous, independent, scientific research, and will be provided following review and approval of a research proposal, Statistical Analysis Plan (SAP), and execution of a Data Sharing Agreement (DSA). Data requests can be submitted at any time after approval in the U.S. and Europe and after acceptance of this manuscript for publication. The data will be accessible for 12 months, with possible extensions considered. For more information on the process or to submit a request, visit the following link: https://vivli.org/ourmember/abbvie/ then select “Home.”

## References

[1] Dahlhamer JM , Zammitti EP, Ward BW, Wheaton AG, Croft JB. Prevalence of inflammatory bowel disease among adults aged ≥18 years–United States, 2015. Morb Mortal Wkly Rep. 2016;65:1166-1169. 10.15585/mmwr.mm6542a3 27787492

[2] Rutgeerts PJ. The limitations of corticosteroid therapy in Crohn’s disease. Aliment Pharmacol Ther. 2001;15:1515-1525. 10.1046/j.1365-2036.2001.01060.x 11563990

[3] Steinhart AH , Ewe K, Griffiths AM, Modigliani R, Thomsen OO. Corticosteroids for maintaining remission of Crohn’s disease. Cochrane Database Syst Rev. 2000;2003:CD000301. 10.1002/14651858.CD000301 10796525 PMC7032673

[4] Lichtenstein GR , Feagan BG, Cohen RD, et al Serious infections and mortality in association with therapies for Crohn’s disease: TREAT registry. Clin Gastroenterol Hepatol. 2006;4:621-630. 10.1016/j.cgh.2006.03.002 16678077

[5] Lewis JD , Scott FI, Brensinger CM, et al Increased mortality rates with prolonged corticosteroid therapy when compared with antitumor necrosis factor-α-directed therapy for inflammatory bowel disease. Am J Gastroenterol. 2018;113:405-417. 10.1038/ajg.2017.479 29336432 PMC5886050

[6] Farraj KL , Pellegrini JR, Munshi RF, et al Chronic steroid use: an overlooked impact on patients with inflammatory bowel disease. JGH Open. 2022;6:910-914. 10.1002/jgh3.12841 36514507 PMC9730704

[7] Zhdanava M , Zhao R, Manceur AM, et al Economic and clinical burden of chronic corticosteroid use in patients with Crohn’s disease initiated on biologic or conventional therapies in the US: a retrospective claims study. J Am Pharm Assoc. 2024;64:386-394.e10. 10.1016/j.japh.2023.11.014 37956768

[8] Siegel CA , Yang F, Eslava S, Cai Z. Treatment pathways leading to biologic therapies for ulcerative colitis and Crohn’s disease in the United States. Clin Transl Gastroenterol. 2020;11:e00128. 10.14309/ctg.0000000000000128 32463619 PMC7145024

[9] Targownik LE , Nugent Z, Singh H, Bernstein CN. Prevalence of and outcomes associated with corticosteroid prescription in inflammatory bowel disease. Inflamm Bowel Dis. 2014;20:622-630. 10.1097/MIB.0000000000000008 24583478

[10] Raine T , Melmed GY, Finney-Hayward T, et al P488 trends in corticosteroid (CS) use over time and following diagnosis in patients with inflammatory bowel disease (IBD), using IBM^®^ MarketScan^®^. J Crohns Colitis. 2022;16:i454-i455. 10.1093/ecco-jcc/jjab232.615

[11] Nancey S , Hébuterne X, Gilletta C, Hacques E, Roblin X. Prevalence of the oral corticosteroid exposure and excessive use in patients with inflammatory bowel disease: data from four French Referral Centers of the International DICE Study. J Clin Med. 2024;13:2652. 10.3390/jcm13092652 38731182 PMC11084465

[12] Torres J , Bonovas S, Doherty G, et al ECCO guidelines on therapeutics in Crohn’s disease: medical treatment. J Crohns Colitis. 2020;14:4-22. 10.1093/ecco-jcc/jjz180 31711158

[13] Lamb CA , Kennedy NA, Raine T, et al British Society of Gastroenterology consensus guidelines on the management of inflammatory bowel disease in adults. Gut. 2019;68:s1-s106. 10.1136/gutjnl-2019-318484 31562236 PMC6872448

[14] Lichtenstein GR , Loftus EV, Isaacs KL, Regueiro MD, Gerson LB, Sands BE. ACG clinical guideline: management of Crohn’s disease in adults. Am J Gastroenterol. 2018;113:481-517. 10.1038/ajg.2018.27 29610508

[15] Panaccione R , Rutgeerts P, Sandborn WJ, Feagan B, Schreiber S, Ghosh S. Review article: treatment algorithms to maximize remission and minimize corticosteroid dependence in patients with inflammatory bowel disease. Aliment Pharmacol Ther. 2008;28:674-688. 10.1111/j.1365-2036.2008.03753.x 18532990

[16] Feuerstein JD , Ho EY, Shmidt E, et al AGA clinical practice guidelines on the medical management of moderate to severe luminal and perianal fistulizing Crohn’s disease. Gastroenterology. 2021;160:2496-2508. 10.1053/j.gastro.2021.04.022 34051983 PMC8988893

[17] Jairath V , Narula N, Ungaro RC, Romo Bautista I, Adsul S. Novel outcomes in inflammatory bowel disease. J Crohns Colitis. 2025;19:1-19. 10.1093/ecco-jcc/jjaf040 PMC12032607 40078047

[18] Schreiber S , Cross RK, Panaccione R, et al Efficacy and safety of risankizumab by baseline corticosteroid use and achievement of corticosteroid—free clinical and endoscopic outcomes in patients with moderately to severely active Crohn’s disease. Aliment Pharmacol Ther. 2024;60:897-906. 10.1111/apt.18184 39054592

[19] Ferrante M , Panaccione R, Baert F, et al Risankizumab as maintenance therapy for moderately to severely active Crohn’s disease: results from the multicentre, randomised, double-blind, placebo-controlled, withdrawal phase 3 FORTIFY maintenance trial. Lancet. 2022;399:2031-2046. 10.1016/S0140-6736(22)00466-4 35644155

[20] Peyrin-Biroulet L , Chapman JC, Colombel J-F, et al Risankizumab versus ustekinumab for moderate-to-severe Crohn’s disease. N Engl J Med. 2024;391:213-223. 10.1056/NEJMoa2314585 39018531

[21] Turner D , Ricciuto A, Lewis A, et al STRIDE-II: an update on the selecting therapeutic targets in inflammatory bowel disease (STRIDE) initiative of the international organization for the study of IBD (IOIBD): determining therapeutic goals for treat-to-target strategies in IBD. Gastroenterology. 2021;160:1570-1583. 10.1053/j.gastro.2020.12.031 33359090

[22] Safroneeva E , Vavricka SR, Fournier N, et al Impact of the early use of immunomodulators or TNF antagonists on bowel damage and surgery in Crohn’s disease. Aliment Pharmacol Ther. 2015;42:977-989. 10.1111/apt.13363 26271358

[23] Ben-Horin S , Novack L, Mao R, et al Efficacy of biologic drugs in short-duration versus long-duration inflammatory bowel disease: a systematic review and an individual-patient data meta-analysis of randomized controlled trials. Gastroenterology. 2022;162:482-494. 10.1053/j.gastro.2021.10.037 34757139

[24] Noor NM , Lee JC, Bond S, et al A biomarker-stratified comparison of top-down versus accelerated step-up treatment strategies for patients with newly diagnosed Crohn’s disease (PROFILE): a multicentre, open-label randomised controlled trial. Lancet Gastroenterol Hepatol. 2024;9:415-427. 10.1016/S2468-1253(24)00034-7 38402895 PMC11001594

[25] Ben-Horin S , Chowers Y. Review article: loss of response to anti-TNF treatments in Crohn’s disease. Aliment Pharmacol Ther. 2011;33:987-995. 10.1111/j.1365-2036.2011.04612.x 21366636

[26] Sharip M , Nishad N, Pillay L, Goordyal N, Goerge S, Subramanian S. Ustekinumab or vedolizumab after failure of anti-TNF agents in Crohn’s disease: a review of comparative effectiveness studies. J Clin Med. 2024;13:2187. 10.3390/jcm13082187 38673459 PMC11050434

[27] D’Haens G , Panaccione R, Baert F, et al Risankizumab as induction therapy for Crohn’s disease: results from the phase 3 ADVANCE and MOTIVATE induction trials. Lancet. 2022;399:2015-2030. 10.1016/S0140-6736(22)00467-6 35644154

[28] Feagan BG , Sandborn WJ, Gasink C, et al Ustekinumab as induction and maintenance therapy for Crohn’s disease. N Engl J Med. 2016;375:1946-1960. 10.1056/NEJMoa1602773 27959607

