# Supplementary material for: Corticosteroid-sparing effects of risankizumab versus ustekinumab in patients with moderately to severely active Crohn’s disease: post hoc results from the phase 3b SEQUENCE trial
Source: J Crohns Colitis. 2026 Jul 21;20(7):jjag104. doi: 10.1093/ecco-jcc/jjag104 (PMC13387155; doi:10.1093/ecco-jcc/jjag104)
Supplement: jjag104_Supplementary_Data [file jjag104_supplementary_data.docx]

# Table S1. Corticosteroid tapering schedule

| **Drug Name** | **Dose** | **Taper Rate** |
| --- | --- | --- |
| Prednisone (or equivalent) | > 10 mg/day  ≤ 10 mg/day | 5 mg/day per week  2.5 mg/day per week |
| Budesonide | ≤ 9 mg/day | 3 mg/day per week |

# Table S2. Overview of treatment-emergent adverse events by baseline corticosteroid use

| **Parameter, n (%)** | **Corticosteroid use** | |  | | **No corticosteroid use** | |  |
| --- | --- | --- | --- | --- | --- | --- | --- |
|  | **Risankizumab** | **Ustekinumab** | **95% CI for Treatment Difference** | | **Risankizumab** | **Ustekinumab** | **95% CI for Treatment Difference** |
|  | **N = 62** | **N = 71** | **Risankizumab vs**  **Ustekinumab** | | **N = 200** | **N = 194** | **Risankizumab vs**  **Ustekinumab** |
| **Any TEAEs** | 52 (83.9) | 59 (83.1) | 0.8 (-11.9, 13.4) | | 171 (85.5) | 160 (82.5) | 3.0 (-4.2, 10.3) |
| Severe TEAE | 9 (14.5) | 17 (23.9) | -9.4 (-22.7, 3.8) | | 33 (16.5) | 34 (17.5) | -1.0 (-8.4, 6.4) |
| Serious TEAEs | 8 (12.9) | 15 (21.1) | -8.2 (-20.9, 4.4) | | 19 (9.5) | 31 (16.0) | -6.5 (-13.0, 0.1) |
| TEAEs leading to study drug discontinuation | 2 (3.2) | 5 (7.0) | -3.8 (-11.2, 3.6) | | 8 (4.0) | 8 (4.1) | -0.1 (-4.0, 3.8) |
| Death | 0 | 0 | 0 | | 0 | 0 | 0 |
| **TEAEs of special interest** |  |  |  | |  |  |  |
| Adjudicated MACE | 0 | 0 | 0 | | 0 | 1 (0.5) | -0.5 (-1.5, 0.5) |
| Extended MACE | 0 | 0 | 0 | | 0 | 1 (0.5) | -0.5 (-1.5, 0.5) |
| Serious infections | 3 (4.8) | 5 (7.0) | -2.2 (-10.2, 5.8) | | 5 (2.5) | 6 (3.1) | -0.6 (-3.9, 2.7) |
| Opportunistic infections (excluding tuberculosis and herpes zoster) | 1 (1.6) | 0 | 1.6 (-1.5, 4.7) | | 0 | 0 | 0 |
| Herpes zoster | 1 (1.6) | 1 (1.4) | 0.2 (-4.0, 4.4) | | 0 | 0 | 0 |
| Malignant tumors | 0 | 0 | 0 | | 1 (0.5) | 1 (0.5) | -0.0 (-1.4, 1.4) |
| NMSC | 0 | 0 | 0 | | 1 (0.5) | 0 | 0.5 (-0.5, 1.5) |
| Malignancies excluding NMSC | 0 | 0 | 0 | | 0 | 1 (0.5) | -0.5 (-1.5, 0.5) |
| Hypersensitivity | 8 (12.9) | 8 (11.3) | 1.6 (-9.5, 12.8) | | 20 (10.0) | 16 (8.2) | 1.8 (-3.9, 7.4) |
| Hepatic events | 1 (1.6) | 3 (4.2) | -2.6 (-8.2, 3.0) | | 17 (8.5) | 11 (5.7) | 2.8 (-2.2, 7.9) |
| Injection site reactions | 2 (3.2) | 1 (1.4) | 1.8 (-3.4, 7.0) | 3 (1.5) | | 5 (2.6) | -1.1 (-3.9, 1.7) |

MACE, major adverse cardiovascular event; NMSC, nonmelanoma skin cancer; TEAE, treatment-emergent adverse event.

No events of active tuberculosis, serious hypersensitivity, or adjudicated anaphylactic reaction.

# Figure S1. Corticosteroid-free clinical, endoscopic, and quality of life outcomes at weeks 24 and 48 of SEQUENCE in all patients


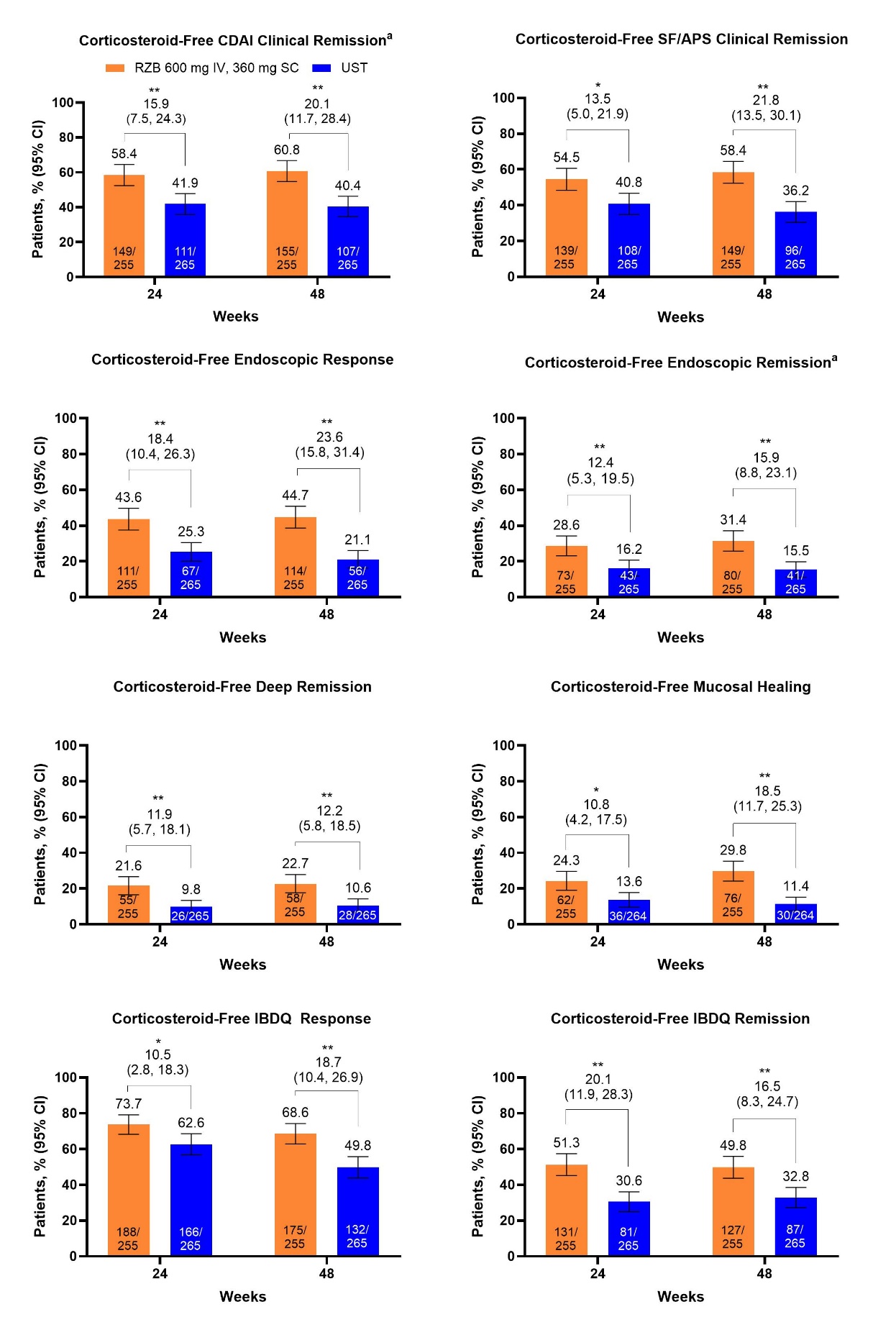


APS, abdominal pain score; CD; Crohn’s disease; CDAI, CD Activity Index; CI, confidence interval; IBDQ, inflammatory bowel disease questionnaire; IV, intravenous; RZB, risankizumab; SC, subcutaneous; SES-CD, Simple Endoscopic Score for CD; SF, stool frequency; UST, ustekinumab.

Values above bars represent % (95% CI).

* *P* ≤ .01; ** *P* ≤ .001. *P* values are nominal.

^a^Corticosteroid-free endoscopic remission and corticosteroid-free clinical remission at week 48 were secondary endpoints of SEQUENCE and were reported previously.

Corticosteroid-free, no corticosteroids at the corresponding study visit; CDAI clinical remission, CDAI < 150; SF/APS clinical remission, average daily SF ≤ 2.8 and daily APS ≤ 1 and both not worse than baseline; endoscopic response, > 50% decrease from baseline in SES-CD or ≥ 2-point reduction from baseline for patients with isolated ileal disease and baseline SES-CD of 4; endoscopic remission, SES-CD ≤ 4 and ≥ 2-point reduction vs baseline and no subscore > 1 in any individual variable; deep remission, CDAI clinical remission plus endoscopic remission; mucosal healing, SES-CD ulcerated surface subscore of 0 in patients with SES-CD ulcerated surface subscore ≥ 1 at baseline as scored by a central reviewer; IBDQ response, increase in IBDQ total score ≥ 16 points from baseline; IBDQ remission, IBDQ total score ≥ 170 points.
